# Neuronal and astrocyte dysfunction diverges from embryonic fibroblasts in the *Ndufs4^fky/fky^* mouse

**DOI:** 10.1042/BSR20140151

**Published:** 2014-11-21

**Authors:** Matthew J. Bird, Xiaonan W. Wijeyeratne, Jasper C. Komen, Adrienne Laskowski, Michael T. Ryan, David R. Thorburn, Ann E. Frazier

**Affiliations:** *Murdoch Childrens Research Institute, Royal Children's Hospital, Parkville, VIC 3052, Australia; †Department of Paediatrics, University of Melbourne, Parkville, VIC 3052, Australia; ‡Department of Biochemistry, La Trobe Institute for Molecular Science, La Trobe University, Bundoora, VIC 3086, Australia; §Victorian Clinical Genetics Services, Royal Children's Hospital, Parkville, VIC 3052, Australia

**Keywords:** metabolic stress, mitochondrial disease, mouse models, neuropathology, primary cells, reactive oxygen species, BN-PAGE, blue native-PAGE, CI–CV, complex I–complex V, CS, citrate synthase, CV, complex V, DHE, dihydroethidium, FCCP, carbonyl cyanide *p*-trifluoromethoxyphenylhydrazone, H_2_O_2_, hydrogen peroxide, HBSS, Hanks buffered saline solution, KO, knockout, LS, Leigh syndrome, MEF, mouse embryonic fibroblast, NBM, neurobasal medium, OXPHOS, oxidative phosphorylation, PI, propidium iodide, RET, reverse electron transfer, ROS, reactive oxygen species, ΔΨ_m_, mitochondrial membrane potential

## Abstract

Mitochondrial dysfunction causes a range of early-onset neurological diseases and contributes to neurodegenerative conditions. The mechanisms of neurological damage however are poorly understood, as accessing relevant tissue from patients is difficult, and appropriate models are limited. Hence, we assessed mitochondrial function in neurologically relevant primary cell lines from a CI (complex I) deficient *Ndufs4* KO (knockout) mouse (*Ndufs4^fky/fky^*) modelling aspects of the mitochondrial disease LS (Leigh syndrome), as well as MEFs (mouse embryonic fibroblasts). Although CI structure and function were compromised in all *Ndufs4^fky/fky^* cell types, the mitochondrial membrane potential was selectively impaired in the MEFs, correlating with decreased CI-dependent ATP synthesis. In addition, increased ROS (reactive oxygen species) generation and altered sensitivity to cell death were only observed in *Ndufs4^fky/fky^* primary MEFs. In contrast, *Ndufs4^fky/fky^* primary isocortical neurons and primary isocortical astrocytes displayed only impaired ATP generation without mitochondrial membrane potential changes. Therefore the neurological dysfunction in the *Ndufs4^fky/fky^* mouse may partly originate from a more severe ATP depletion in neurons and astrocytes, even at the expense of maintaining the mitochondrial membrane potential. This may provide protection from cell death, but would ultimately compromise cell functionality in neurons and astrocytes. Furthermore, RET (reverse electron transfer) from complex II to CI appears more prominent in neurons than MEFs or astrocytes, and is attenuated in *Ndufs4^fky/fky^* cells.

## Abstract 2

We describe how the metabolic response in neurologically relevant primary astrocytes and neurons diverges from that of mouse embryonic fibroblasts isolated from the *Ndufs4^fky/fky^* mouse model of mitochondrial disease.

## INTRODUCTION

Mitochondrial diseases are a collection of rare disorders, each featuring mitochondrial dysfunction, estimated to affect 1 in 5000 live births [1]. Presentation of the disease is diverse, potentially involving any tissue, with any severity and at any age [2]. Typically however, there is involvement of the more metabolically active tissues displaying high-energy demand, notably brain [3,4]. The genetic basis of the disease is also complex, with pathogenic mutations occurring in over 100 genes required to make a functional mitochondrion, including the OXPHOS (oxidative phosphorylation) system [4,5]. Although mutations in disease related OXPHOS genes are associated with a broad range of classical mitochondrial disorders, one of the most common of these is LS (Leigh syndrome) [4].

LS is a progressive neurodegenerative disorder where patients exhibit a broad range of neurologically based symptoms including ataxia, breathing abnormalities, optic atrophy, developmental retardation, hearing impairment and seizures [6–8]. Pathologically, the disease is characterized by symmetrical bilateral lesions in the basal ganglia, thalamus, or brain stem, increased lactate in the blood and/or cerebral spinal fluid, gliosis and neuronal demyelination [6–8]. Although the gross biochemical and clinical manifestations of LS are well characterized, the difficulty in obtaining fresh patient samples from neurological regions of interest has limited our understanding of the biochemical features at a cellular level.

To this end, a number of animal and cellular model systems of primary mitochondrial dysfunction have recently been generated (reviewed in [9,10]), including the *Ndufs4^fky/fky^* and *Ndufs4* KO (knockout) mice lacking the OXPHOS CI (complex I) subunit NDUFS4 [11,12]. Like LS patients, the NDUFS4 deficient mice develop hyperintense bilateral lesions of the brain stem [13,14]. The mice also exhibit clinical manifestations similar to those observed in LS patients [11–14]. Moreover, the brain-specific *Ndufs4* KO mouse is nearly biochemically and phenotypically indistinguishable from the whole animal KO, confirming the neurological basis of the disease and its clinical features [13]. As the NDUFS4 deficient mice have recapitulated many of the common features of LS, these mice provide the opportunity to examine how mitochondrial systems and dynamics are affected in a neurological setting.

So far, analysis of whole-brain preparations from NDUFS4-deficient mice [12,13] has revealed that, like NDUFS4-deficient patients and mouse fibroblasts [15,16], CI structure and function is disrupted. In each system, CI forms an ≈830 kDa crippled complex on BN-PAGE (blue native-PAGE). Furthermore, the CI defect was found to affect CI-dependent ATP synthesis in isolated brain mitochondria [12]. There is also evidence of oxidative damage to proteins in the olfactory bulb of NDUFS4-deficient mice, one of the areas of the brain affected by the disease [13]. The latter is of particular interest considering that NDUFS4-deficient patient and adult mouse fibroblasts produce elevated amounts of ROS (reactive oxygen species), including superoxide (O_2_^•−^) and H_2_O_2_ (hydrogen peroxide) [17–19]. It therefore seems reasonable to expect that disruption of CI in neurologically relevant cell types will also result in increased production of ROS, thus contributing to neuropathogenesis. However, in *Ndufs4* KO mouse primary mesencephalic neurons, O_2_^•−^ production in isolated mitochondria, and H_2_O_2_ production in whole cells was normal relative to controls [20,21].

Accordingly, characterizing cell-type-specific mechanisms of disease, such as ROS dynamics, will increase our understanding of the modes of disease pathogenesis in LS. To this end, we sought to isolate and characterize primary isocortical astrocytes and isocortical neurons from the *Ndufs4^fky/fky^* mouse. Despite astrocytes being the most abundant cell type in the mammalian brain [22,23], their role in mitochondrial disease is often overlooked. Astrocytes are metabolically intricately linked to neurons for proper cellular function through processes such as neurotransmitter recycling and extracellular potassium homoeostasis [23,24]. Furthermore, they have been implicated in disease progression in both LS patients and NDUFS4-deficient mice, where an infiltration of activated astrocytes into affected brain regions is a feature in both systems [7,13]. In conjunction, we also investigated mitochondrial function in *Ndufs4^fky/fky^* MEFs (mouse embryonic fibroblasts), which serve as a control cell type from a non-neurological tissue for our studies. Our examination of the OXPHOS system and associated mitochondrial processes in these primary *Ndufs4^fky/fky^* astrocytes, neurons and MEFs thus provided the opportunity to examine disease mechanisms at a cell-type-specific level.

## EXPERIMENTAL

### Mouse model

The *Ndufs4^fky/fky^* mouse was generated by the spontaneous insertion of the B2 SINE (Short Interspersed Nuclear Element) into the *NADH dehydrogenase (ubiquinone) Fe–S protein 4 (Ndufs4)* gene as previously described [12], and maintained on a BALB/c background. Heterozygous mice were bred for the generation of pups and embryos according to an approved protocol by the Murdoch Childrens Research Institute Animal Ethics Committee (A662). Mice were housed on 12 h light/dark cycles and provided *ad libitum* access to food and water.

### Cell culture methods

Primary isocortical astrocytes and isocortical neurons were isolated from individual 3–5-day-old pups and 17–18-day post-fertilization embryo isocortices, respectively, as previously described [25,26]. Briefly, tissues were collected in HBSS (Hanks buffered saline solution) without Mg^2+^ and Ca^2+^ (Gibco), and digested in 0.6% (w/v) trypsin with 2.78 mM glucose [30 min, 37°C, 5% (v/v) CO_2_]. Following trypsin neutralization with 1 mg/ml soya trypsin inhibitor (Invitrogen) containing 100 units/ml DNAse I (Invitrogen), cells were mechanically dissociated and diluted in HBSS containing Mg^2+^ and Ca^2+^. Isolated astrocytes were diluted in DMEM containing 25 mM glucose (abbreviated in text as ‘glucose’ medium; Thermo scientific) supplemented with 10% (v/v) FCS, and plated in flasks pre-coated with 50 μg/ml poly D-lysine. At confluency, astrocyte cultures were shaken at 200 rpm, 37°C for 14–16 h to remove contaminating cell types. Isolated neurons were diluted in NBM (neurobasal medium; Invitrogen) supplemented with B-27 (Invitrogen), 292 mg/l L-glutamine, and plated in flasks pre-coated with 50 μg/ml poly D-lysine, or coverslips pre-coated with both 50 μg/ml poly D-lysine and 10 μg/ml laminin. The purity of neuronal and astrocyte cultures was assessed by immunocytochemistry (Supplemental Materials and methods section and Supplementary Figure S1).

Primary MEFs were isolated from day 12.5–14.5 post-fertilization embryos. After the removal of the head and visceral organs, tissues were disrupted mechanically and subsequently digested with 0.125% trypsin (30 min, 37°C, 5% CO_2_). Cells were then mechanically dissociated, pelleted, and plated in glucose medium in culture flasks coated with 0.1% gelatin.

Cells were maintained at 37°C with 5% CO_2_ in a humidified chamber. All cell isolation buffers (HBSS) and culture media contained 60.3 mg/l penicillin and 100 mg/l streptomycin. mtDNA (mitochondrial DNA) deficient (ρ°) and control (ρ^+^) mouse LM(TK^−^) cells [27] were maintained on glucose medium (described above) with 50 μg/ml uridine. Where described, astrocytes and MEFs were alternatively cultured in DMEM containing 5 mM galactose (abbreviated in text as ‘galactose’ medium; Gibco) supplemented with 10% dialysed FCS and 292 mg/l L-glutamine.

### Mitochondrial isolation

Mitochondria were isolated from primary cells as previously described [28].

### CI and CS (citrate synthase) activity

CI and CS activities in primary cell cultures were determined relative to total protein at 30°C as previously described [29]. Briefly, cells were homogenized in buffer (200 mM mannitol, 70 mM sucrose, 5 mM HEPES free acid, 1 mM EGTA, pH 7.2) with a Teflon-glass homogenizer in combination with a benchtop drill at 1000 rpm. Debris was removed by a low speed spin at 600 ***g***. For CI activity measurements, the samples were diluted in buffer containing CoenzymeQ_1_, NADH, potassium cyanide and antimycin A in the presence or absence of rotenone. The activity of CI was measured in a spectrophotometer as the rotenone-sensitive rate of NADH oxidation at 340 nm over 3 min. For CS activity measurements, sample was diluted in buffer containing 5,5′-dithio-bis-(2-nitrobenzoic acid), oxaloacetate and acetyl CoA. The rate of thionitrobenzoate anion production was monitored in a spectrophotometer over 3 min at 412 nm.

### Western blot analysis

Primary cell pellets were solubilized in RIPA buffer [50 mM Tris/HCl, 150 mM NaCl, 1% (v/v) Triton X-100, 1% (w/v) Na deoxycholate, 0.1% (w/v) SDS, 1 mM EDTA, pH to 7.4] containing protease inhibitor (Roche), diluted in LDS (lithium dodecyl sulfate) sample buffer (Invitrogen), heat denatured (10 min, 60°C) and separated on 10% Novex bis-tris gels (Invitrogen). Gels were transferred to 0.45 μm PVDF membrane (Millipore) for immunodecoration. Membranes were probed with anti-NDUFS4 (MitoSciences, MS104) or anti-SDHA (Molecular Probes, A-11142).

### BN-PAGE

Cell pellets were solubilized in either 1% digitonin or 1% Triton X-100 and protein complexes separated by BN-PAGE as described [30]. Briefly, 50–80 μg of whole cell lysate was separated per lane on 4–10 or 4–13% BN-PAGE gels (Invitrogen). Gels were transferred to PVDF membrane (Millipore) for immunodecoration. Membranes were probed with antibodies against the CI subunit NDUFA9 [31], CII subunit SDHA (Invitrogen) or complex III subunit Core1 (Invitrogen).

### ATP synthesis assay

ATP synthesis rates were measured in technical duplicates in primary cell cultures and isolated mitochondria as described [32]. Briefly, 10 μg of cells, or 2 μg isolated mitochondria were diluted 10-fold in ATP synthesis buffer containing 40–80 μg/ml digitonin (whole cells only) and substrates with/without inhibitors [succinate (10 mM); malonate (1 mM); glutamate (10 mM); pyruvate (10 mM); malate (10 mM); rotenone (2.5 μM)] and incubated at 37°C for 20 min. Reactions were stopped with perchloric acid on ice, neutralized with potassium hydroxide and MOPS. ATP concentration in samples was determined using a luciferase-based assay (Roche, 11699695001) in a microplate reader (BMG Labtech, FLUOstar OPTIMA). Rates are reported as the ratio to measurements made with succinate+rotenone.

### Membrane potential measurements

Cells were cultured in black 96-well plates (2×10^4^ cells/well for primary astrocytes and MEFs, 5×10^4^ cells/well for ρ° and ρ^+^ LM(TK^−^) cells) and incubated with media containing 75 nM tetramethylrhodamine methyl ester (TMRM; Sigma) and 2 μg/ml Hoechst-33258 (Sigma) (40 min, 37°C, 5% CO_2_). Subsequently, cells were washed with PBS before the ΔΨ_m_ (mitochondrial membrane potential) was determined in a minimum of three wells as the ratio of TMRM to Hoechst fluorescence (BMG Labtech, FLUOstar OPTIMA). Cells treated with the CI inhibitor rotenone (2.5 μM) or the protonophore FCCP (carbonyl cyanide *p*-trifluoromethoxyphenylhydrazone); Sigma; 20 μM) were used as controls.

Alternatively, the ΔΨ_m_ was determined by microscopy in cells cultured in glass bottom dishes (WPI, FD-35-100). Cells were incubated in HBSS with Mg^2+^ and Ca^2+^ containing 37.5 nM TMRM (30 min, 37°C, 5% CO_2_). Cells were visualized at 37°C on a DeltaVision OMX V3 Imaging System (Applied Precision) using the TRITC (tetramethylrhodamine β-isothiocyanate) filter set with an oil-immersion objective (Olympus, IX71), fitted with a CoolSNAP HQ^2^ camera (Photometrics). After initial imaging, either 20 μM FCCP or 2.5 μM rotenone was added to dishes and regions of interest re-captured. Images were de-convoluted, cropped and projected with maximum intensity (Applied Precision, SoftWoRx v5.5). Mitochondrial rich perinuclear regions were selected in greater than 60 cells per dish and average TMRM fluorescence determined pre- and post-FCCP or rotenone application.

### Superoxide (O_2_^•−^) detection

Cells were cultured in black 96-well plates (2×10^4^ cells/well) and incubated with media containing 10 μg/ml DHE (dihydroethidium; Sigma) and 2 μg/ml Hoechst-33258 (40 min, 37°C, 5% CO_2_). Cells were washed in PBS, and the rate of O_2_^•−^ production determined in a minimum of three wells as the ratio of DHE to Hoechst fluorescence (BMG Labtech, FLUOstar OPTIMA). Cells treated with the CI inhibitor rotenone (2.5 μM) were used as a control.

### H_2_O_2_ detection

The rate of H_2_O_2_ production was measured as previously described [33] in 5 μg isolated mitochondria using the fluorescent probe Amplex Red with the substrate and inhibitor combinations detailed in the text [succinate (10 mM); malonate (1 mM); glutamate (10 mM); pyruvate (10 mM); malate (10 mM); rotenone (2.5 μM)]. H_2_O_2_ production was measured fluorometrically over a 20 min period (BMG Labtech, FLUOstar OPTIMA).

### Cell death assays

Where described, cells were treated with 50 μM H_2_O_2_ in culture media for 6 h. Cells were harvested using trypsin, and diluted to 2×10^5^ cells/ml in annexin-V binding buffer (10 mM Hepes, 140 mM NaCl, 2.5 mM CaCl_2_, pH to 7.4). An aliquot of 500 μl was co-incubated with 5 μl annexin-V (Invitrogen) and 1 μg/ml PI (propidium iodide, Sigma) for 10 min at room temperature then analysed by flow cytometry (Becton Dickinson, LSR II flow cytometer) with the FITC-A and PI-488-695/40-A lasers. Viable cells were distinguished from dead cells using the forward- and side-scatter parameters (De Novo, FCS express V4). Healthy cells were considered as annexin-V and PI double negative, apoptotic cells as annexin-V positive, and necrotic cells as annexin-V and PI double positive. Gating strategy is depicted in Supplementary Figures S2(A) and S2(B).

### Statistics

Unless otherwise described, error bars represent standard error of the mean (S.E.M.) and *P* values are generated from two-tailed unpaired *t* tests (GraphPad, PRISM V6.0b) and reported when ≤0.05.

## RESULTS

### CI activity is equally reduced in *Ndufs4^fky/fky^* cell lines

**Figure 1:**
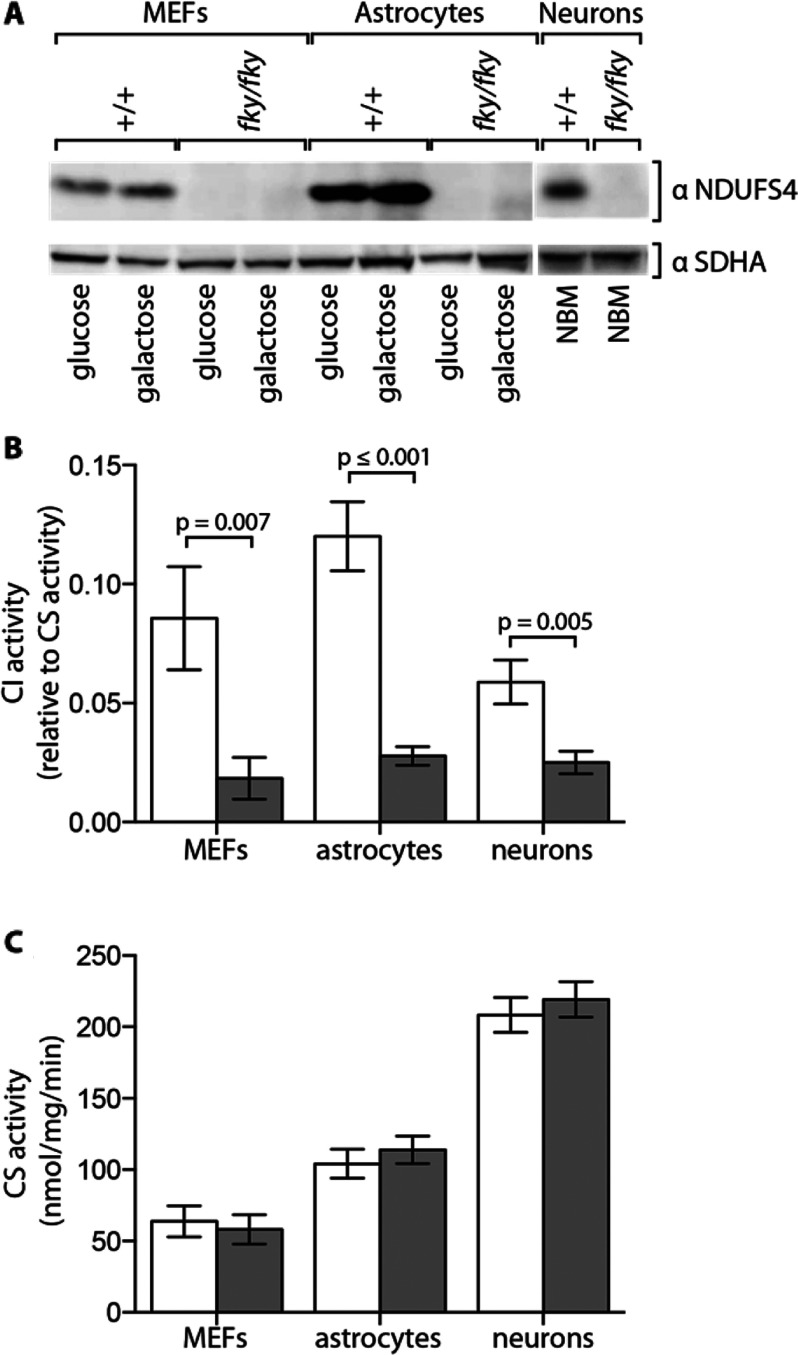
CI activity in *Ndufs4^fky/fky^* cell lines Expression of the CI subunit NDUFS4 was examined in cell lysates that were prepared from primary cell cultures from +/+ (white bars) and *Ndufs4^fky/fky^* (grey bars) mice that were grown in either glucose or galactose medium (primary MEFs, primary astrocytes), or NBM (primary neurons) by Western blot analysis using antibodies against the CI subunit NDUFS4 and the CII subunit SDHA as a loading control (**A**). Images have been digitally cropped for clarity. CI activity relative to CS activity (**B**) and CS activity (**C**) were measured in primary cell culture lysates from +/+ and *Ndufs4^fky/fky^* mice maintained on either glucose medium (primary MEFs, primary astrocytes) or NBM (primary neurons) by spectrophotometry. Error bars=S.E.M., *n*≥7, *P* values generated from two-tailed unpaired *t* tests. CI, complex I; CII, complex II; CS, citrate synthase.

The NDUFS4 subunit was undetectable in all *Ndufs4^fky/fky^* primary cell types examined (Figure 1A), consistent with the systemic disruption of NDUFS4 in the mouse and in keeping with previous reports on all tissues studied [12]. This was not changed by culturing *Ndufs4^fky/fky^* primary MEFs and astrocytes under conditions that favour ATP generation by the OXPHOS system (on galactose). CI activity was severely impaired in *Ndufs4^fky/fky^* primary MEFs (21% of +/+), astrocytes (23% of +/+) and neurons (42% of +/+) under standard culture conditions (on glucose) (Figure 1B), similar to reports on mouse tissues [12]. To account for alterations in mitochondrial volume and/or number, CI activity was normalized to CS activity, which was not affected by loss of NDUFS4 (Figure 1C). It is worth noting that while the activity of CI relative to CS was similar in each of the *Ndufs4^fky/fky^* cell types, the activity of CI relative to CS in +/+ cells was lower in neurons than in astrocytes and MEFs (Figure 1B).

Taken together with previous data obtained from the *Ndufs4^fky/fky^* mice [12], it appears unlikely that a tissue-specific difference in CI activity is the basis for the predominant neurological dysfunction in the *Ndufs4^fky/fky^* mouse.

### CI appears as a crippled complex in *Ndufs4^fky/fky^* cell lines

**Figure 2:**
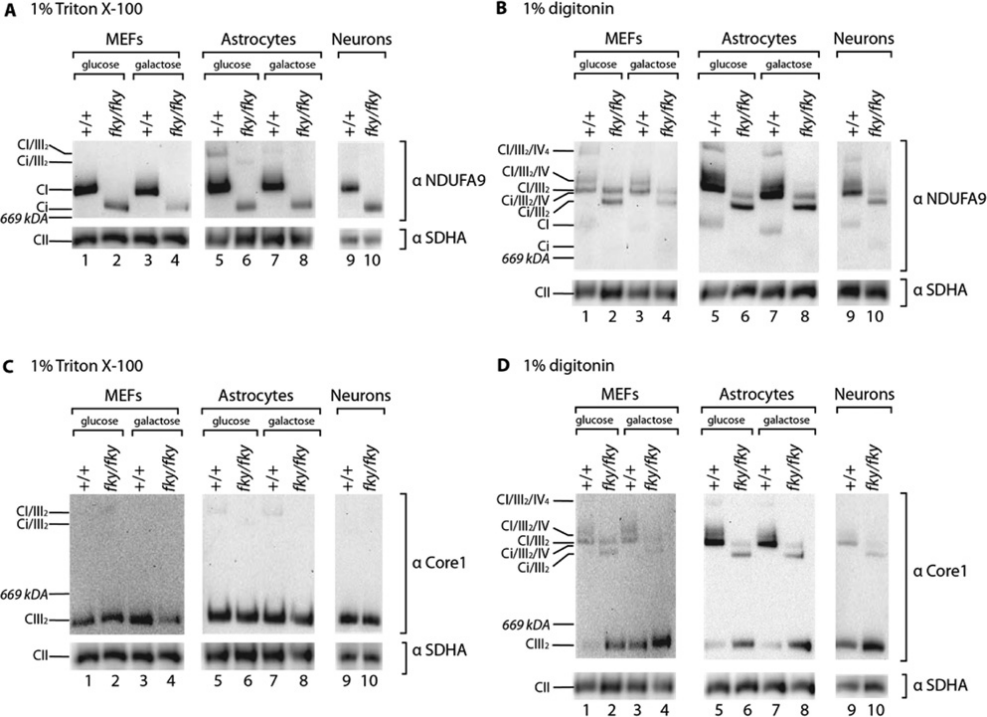
BN-PAGE analyses of respiratory complexes in *Ndufs4^fky/fky^* cell lines Cells derived from +/+ and *Ndufs4^fky/fky^* mice maintained on either glucose or galactose medium (primary MEFs, primary astrocytes), or NBM (primary neurons), were solubilized in buffer containing either 1% Triton X-100 (A and C) or 1% digitonin (**B**, **D**), and protein complexes separated by BN-PAGE. Gels were transferred to membrane and immunodecorated with antibody targeted to the CI subunit NDUFA9 (**A**, **B**), or targeted to the CIII subunit Core1 (**C**, **D**). CII subunit SDHA was used as a loading control. *n*=1–3. Images have been digitally cropped for clarity. CI–IV, complexes I–IV; Ci, crippled CI formed in the absence of NDUFS4.

We next investigated the assembly status of CI in the primary cell lines. Consistent with previous reports, using BN-PAGE we observed that CI lacking NDUFS4 forms a crippled assembly intermediate (Ci) of ≈830 kDA and is reduced in abundance when cells were solubilized in Triton X-100 to isolate individual OXPHOS complexes (Figure 2A: +/+, odd lanes; *Ndufs4^fky/fky^*, even lanes) [12,15,16,34]. This was independent of culture conditions for all primary cells (Figure 2A). Furthermore, when *Ndufs4^fky/fky^* cell pellets were solubilized in the milder detergent digitonin to retain supramolecular interactions with other OXPHOS complexes (Figures 2B and 2D: +/+, odd lanes; *Ndufs4^fky/fky^*, even lanes), supercomplexes appeared at smaller molecular weights corresponding to supercomplexes containing the crippled CI. Again, this was observed independent of culture conditions for all primary cells (Figures 2B and 2D).

In addition, the level of mature complex III (CIII_2_) was similar in all cells and under all culture conditions examined (Figure 2C). However the abundance of free CIII_2_ not bound in supercomplexes appeared to be increased in *Ndufs4^fky/fky^* cells (Figure 2D), consistent with reduced abundance of supercomplex species containing crippled CI.

Together these data suggest that the assembly of CI itself and supercomplexes containing crippled CI is equally disrupted in detergent solubilized cell pellets across all primary *Ndufs4^fky/fky^* cell types and culture conditions. Accordingly, these data do not provide any further insight into the mechanisms behind the tissue-specific nature of the disease, nor suggest any adaptive changes in MEFs and astrocytes on galactose in response to a greater dependence on OXPHOS for cellular ATP production.

### The ΔΨ_m_ is selectively impaired in *Ndufs4^fky/fk^*^y^ primary MEFs

**Figure 3:**
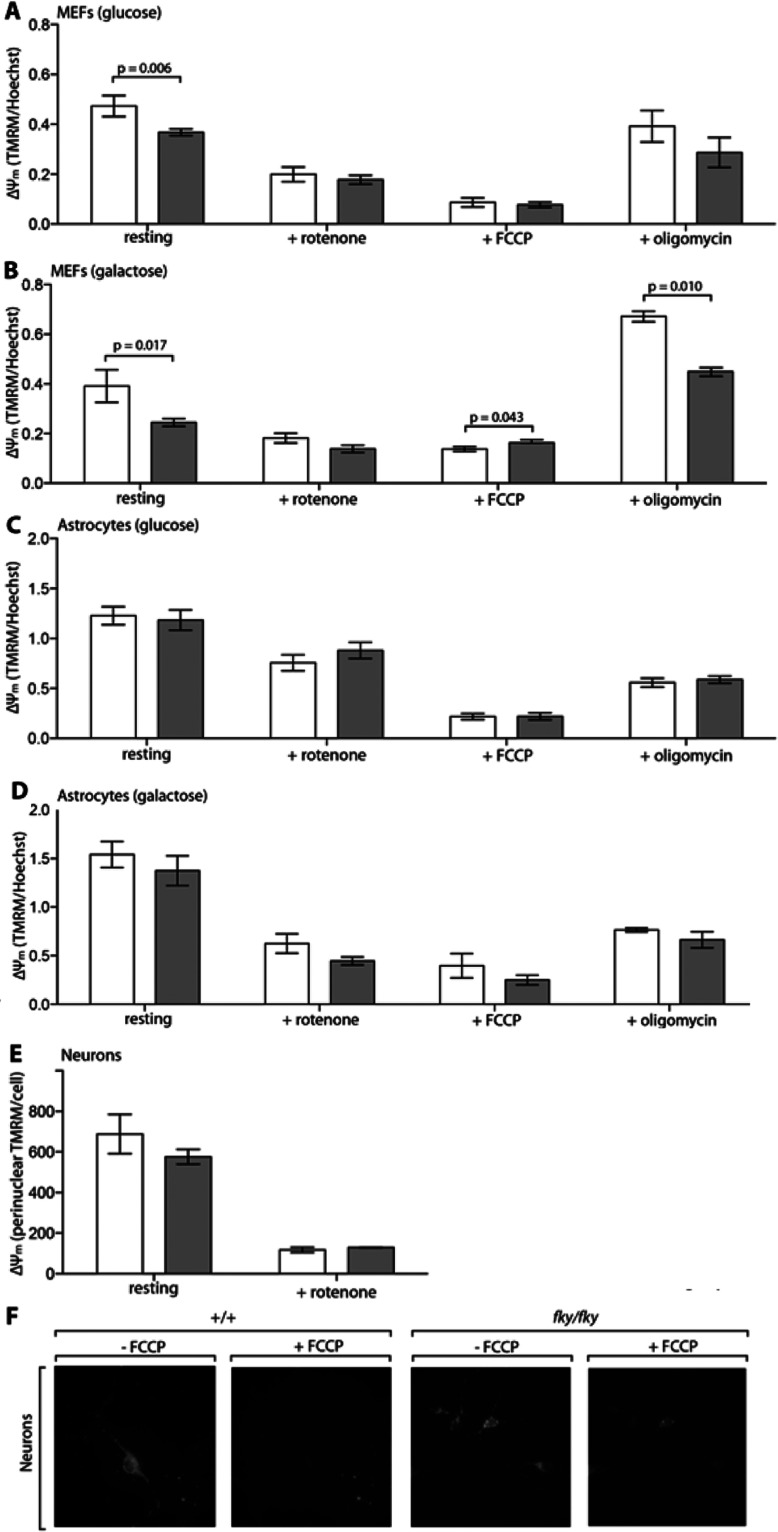
Analysis of the ΔΨ_m_ in *Ndufs4^fky/fky^* cell lines The ΔΨ_m_ was measured by the accumulation of TMRM in mitochondria and made relative to cell number in primary MEFs and astrocytes from +/+ (white bars) and *Ndufs4^fky/fky^* (grey bars) mice, maintained on glucose (**A**, **C**) or galactose (**B**, **D**) medium. The ΔΨ_m_ was reduced with the CI inhibitor rotenone or the protonophore FCCP, or modulated by the CV inhibitor oligomycin, as indicated. The ΔΨ_m_ was assessed by microscopy in primary neurons (**E**) pre- and post-incubation with rotenone, by measuring the accumulation of the cationic dye TMRM in the mitochondrial-rich perinuclear region. Greater than 60 cells per dish were analysed using ≥2 cell cultures. Representative images are shown for primary neurons in (**F**), pre- and post-incubation with FCCP. Error bars=S.E.M., *n*≥3 (**A**–**D**), *P* values generated from two-tailed unpaired *t* tests. CI and CV, complexes I and V; ΔΨ_m_, mitochondrial membrane potential.

As noted above, CI activity and assembly were severely impaired in all *Ndufs4^fky/fky^* primary cell types studied. Considering the integral nature of CI to proton pumping across the IMM, it might be expected that this could affect the ΔΨ_m_. Moreover, culturing cells on galactose increases the dependence on OXPHOS, which could further impact the ΔΨ_m_. Nonetheless, under basal conditions, the ΔΨ_m_ was significantly impaired only in *Ndufs4^fky/fky^* primary MEFs (Figures 3A and 3B), while it appeared normal in astrocytes (Figures 3C and 3D) and neurons (Figures 3E and 3F). More specifically, the ΔΨ_m_ at rest in *Ndufs4^fky/fky^* primary MEFs on glucose medium was depolarized (more electroneutral; Figure 3A; 75% of +/+), and appeared further depolarized on galactose medium (Figure 3B; 60% of +/+). As control experiments, we found that the ΔΨ_m_ was equally depolarized in all cell types under all culture conditions following application of the CI inhibitor rotenone, or the protonophore FCCP (Figures 3A–3F). In addition, methodological approaches were validated using mouse fibroblast ρ° cells, which do not possess a functional OXPHOS system [27]. In the ρ° cells at rest, the ΔΨ_m_ was clearly depolarized when measured by both plate reader (Supplementary Figure S3A; 62% of ρ^+^) and microscopy (Supplementary Figure S3B and S3C; 48% of ρ^+^), consistent with previous observations in similar cells [35,36].

Primary MEFs and astrocytes did however respond differently to the application of the CV (complex V) inhibitor oligomycin. Inhibition of CV blocks the flow of electrons back into the mitochondrial matrix, which should result in hyperpolarization (more electronegative) of the ΔΨ_m_ when OXPHOS is in action. In primary MEFs cultured on glucose medium (Figure 3A), oligomycin had no effect on the ΔΨ_m_, yet it hyperpolarized the ΔΨ_m_ when cells were cultured on galactose (Figure 3B). In contrast, in primary astrocytes the ΔΨ_m_ was depolarized after applying oligomycin irrespective of culture media (Figures 3C and 3D). The phenomenon was not genotype specific though, and could reflect toxicity of the oligomycin to primary astrocytes.

### CI-dependent ATP synthesis capacity is reduced in *Ndufs4^fky/fky^* primary astrocytes and neurons, but only impaired in primary MEFs when cultured on galactose

The ΔΨ_m_ is utilized to drive a number of mitochondrial processes. One of the most critical of these, and considered to be the core function of mitochondria, is ATP synthesis via CV [37]. In this context, we sought to determine the maximal capacity of CI-dependent ATP synthesis in permeabilized *Ndufs4^fky/fky^* primary cells under optimal substrate conditions. These measurements were obtained in digitonin permeabilized *Ndufs4^fky/fky^* primary cells provided with the CI-dependent substrates glutamate+malate or pyruvate+malate, with or without the CI inhibitor rotenone. All rates were made relative to the CII-dependent rate determined using succinate+rotenone, since these should not be affected by CI deficiency. Importantly, no statistically significant differences were detected between genotypes for rates of CII-dependent ATP synthesis (Table 1).

**Table 1:** CII-dependent rate of ATP synthesis in isolated mitochondria and permeabilized cells from *Ndufs4^fky/fky^* primary cell lines *Rate of ATP synthesis measured using the substrate succinate with the complex I inhibitor rotenone±S.E.M. (*n*). No statistically significant differences were detected (*P*<0.050 level) between +/+ and *fky*/*fky* samples, measured by unpaired *t* tests, correcting for multiple comparisons using the Holm–Sidak method (GraphPad, PRISM V6.0b).

			ATP (μmol/g/min)*	
Primary cell line	Medium	Preparation	+/+	*fky/fky*
MEFs	Glucose	Permeabilzed cells	36±5.1 (9)	37±5.0 (13)
	Galactose	Permeabilized cells	26±4.1 (7)	19±4.6 (8)
	Glucose	Isolated mitochondria	61±12 (6)	65±11 (6)
Astrocytes	Glucose	Permeabilized cells	61±6.1 (13)	68±7.6 (11)
	Galactose	Permeabilized cells	48±3.9 (7)	45±1.4 (5)
	Glucose	Isolated mitochondria	84±11 (3)	89±22 (4)
Neurons	NBM	Permeabilized cells	24±1.7 (17)	29±2.3 (21)

**Figure 4:**
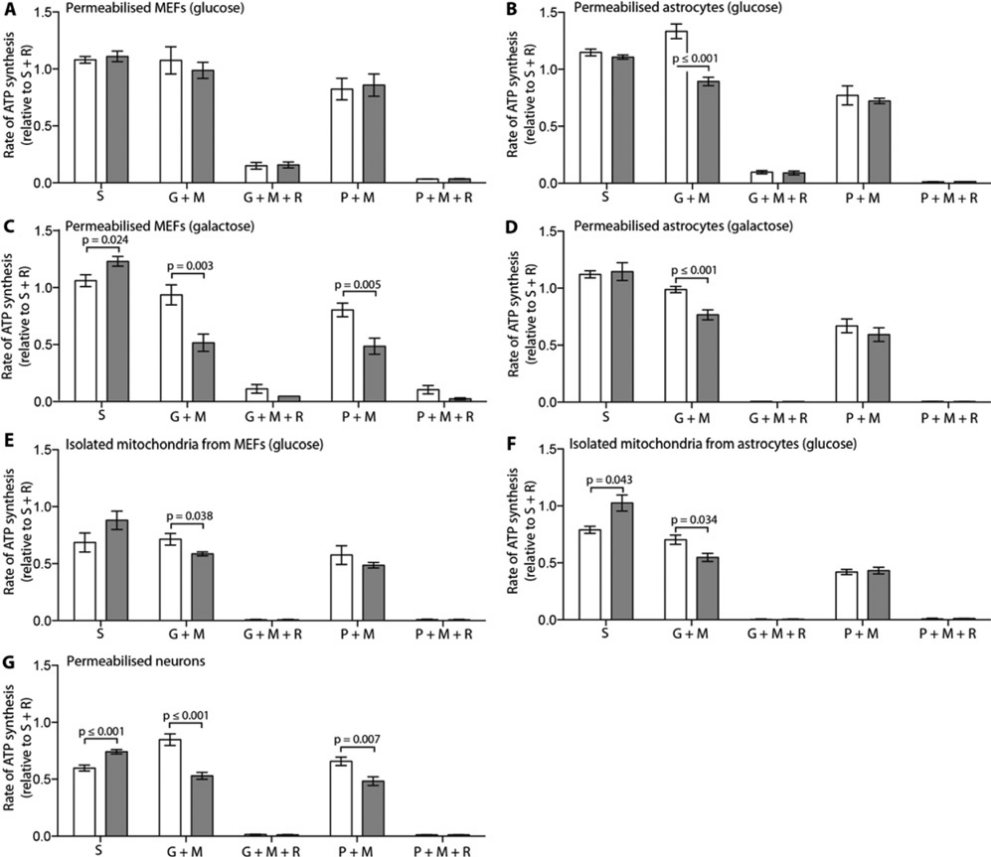
Rates of ATP synthesis in *Ndufs4^fky/fky^* cell lines, expressed relative to the CII-dependent rate determined using succinate+rotenone (S+R) The CII- (S) and CI-dependent (G+M, P+M) rates of ATP synthesis were measured in permeabilized cells or isolated mitochondria from MEFs and astrocytes maintained on glucose (**A**, **B**, **E**, **F**) or galactose (**C**, **D**), and neurons maintained on NBM medium (**G**) from +/+ (white bars) and *Ndufs4^fky/fky^* (grey bars) mice. Where indicated, rotenone was added to inhibit CI. Error bars=S.E.M. Replicates: S (*n*≥3); G+M (*n*≥3); G+M+R (*n*≥2); P+M (*n*≥3); and P+M+R (*n*≥2). *P* values generated from two-tailed unpaired *t* tests. CI, complex I; G, glutamate; M, malate; P, pyruvate; R, rotenone; S, succinate.

In *Ndufs4^fky/fky^* primary MEFs, the CI-dependent ATP synthesis rates were almost indistinguishable from controls on glucose medium (Figures 4A and 4E; 82–105% of +/+) but reduced on galactose (Figure 4C; 55–60% of +/+). In contrast, in *Ndufs4^fky/fky^* primary astrocytes the CI-dependent rate was reduced with glutamate+malate (Figures 4B, 4D and 4F; 67–79% of +/+ on glucose and 77% of +/+ on galactose), but normal with pyruvate+malate irrespective of culture media (Figures 4B, 4D and 4F). In *Ndufs4^fky/fky^* primary neurons the rate of ATP synthesis was reduced with all CI-dependent substrates (Figure 4G; 63–74% of +/+). To confirm the specificity of the substrates, we determined that the CI-dependent rate of ATP synthesis was almost completely abolished in the presence of the CI inhibitor rotenone in all *Ndufs4^fky/fky^* primary cell types under all culture conditions (Figures 4A–4G).

We also noted in permeabilized primary neurons (Figure 4G) that the rate of ATP synthesis with succinate alone relative to the rate with succinate+rotenone was lower (<1), compared with permeabilized MEFs or astrocytes (Figures 4A–4D). Moreover, this relative rate was more reduced in +/+ than *Ndufs4^fky/fky^* neurons (Figure 4G; 0.60 versus 0.74). In addition, a relative rate of <1 is observed in isolated mitochondria from +/+ MEFs and astrocytes, but not *Ndufs4^fky/fky^* cells (Figures 4E and 4F). Together these data imply that RET (reverse electron transfer) may occur in all primary cell types examined. The term RET describes the process in which ubiquinol generated by CII donates electrons to a reverse CI reaction rather than to the forward CIII reaction [38]. RET is blocked by rotenone inhibition of CI. The data also suggest that RET is partially blocked in the *Ndufs4^fky/fky^* primary cells, most prominently in neurons. This is presumably because the CI defect blocks both forward and reverse electron transfer by CI. In addition, these observations indicate that a small amount of residual CI-dependent substrate in our permeabilized cells that is not present in isolated mitochondria may artificially increase the CII-dependent rate of ATP synthesis. Indeed, while the general rates of ATP synthesis were very low when no substrate was provided (Supplementary Figures S4A–S4G), a slightly higher rate was observed in the permeabilized cells compared with the isolated mitochondria and neurons. As a control, we determined that the rates of CII-dependent ATP synthesis in all cell types were sensitive to the CII inhibitor malonate (Supplementary Figures S4A–S4G; rate relative to succinate+rotenone<1).

### Rates of ROS generation are selectively elevated in *Ndufs4^fky/fky^* primary MEFs

Dysfunction of CI may affect not only the ΔΨ_m_ and associated ATP synthesis, but may also be associated with increased ROS production. ROS have the potential to cause oxidative damage to cellular structures, including DNA, proteins and lipids, if not rapidly detoxified by cells [39,40]. As such, we sought to quantify the amount of O_2_^•−^ produced by *Ndufs4^fky/fky^* primary MEFs and astrocytes cultured on either glucose or galactose media, using the O_2_^•−^ specific probe DHE. Additionally, CI- and CII-dependent H_2_O_2_ production was measured in isolated mitochondria from MEFs and astrocytes grown on the glucose medium using Amplex Red. Owing to limitations of sample availability, we were unable to perform comparable measurements of O_2_^•−^ and H_2_O_2_ production in *Ndufs4^fky/fky^* primary neurons.

**Figure 5:**
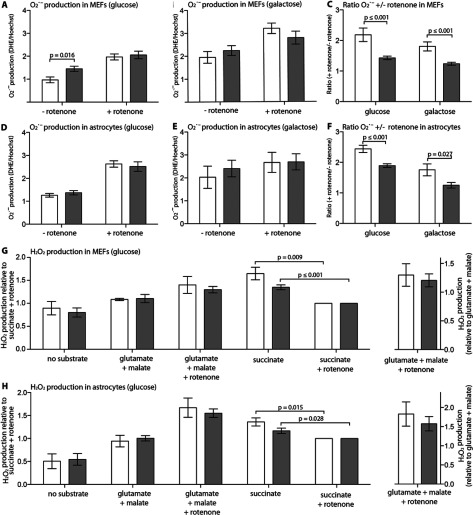
O_2_^•−^ and H_2_O_2_ production in *Ndufs4^fky/fky^* cell lines The rate of O_2_^•−^ production was measured by the cellular accumulation of DHE and made relative to the total cell number in primary MEFs and astrocytes from +/+ (white bars) and *Ndufs4^fky/fky^* (grey bars) mice, maintained on glucose (**A**, **D**), or galactose (**B**, **E**) medium. The rate of O_2_^•−^ production was modulated with the CI inhibitor rotenone, as indicated. The ratio of O_2_^•−^ production from rotenone treated cells to non-treated cells (+ rotenone/- rotenone) is presented in (**C**) and (**F**). Rates of H_2_O_2_ production were measured with Amplex Red in isolated mitochondria from glucose-grown primary MEFs and astrocytes from +/+ and *Ndufs4^fky/fky^* mice (**G**, **H**) and results are displayed either relative to succinate+rotenone (left *y*-axis) or glutamate+malate (right *y*-axis). To maintain statistical power, comparisons have only been made between genotypes with the exception of between groups comparing succinate with or without rotenone, given this difference is of particular interest (**G**, **H**). Error bars=S.E.M., *n*≥3, *P* values generated from two-tailed unpaired *t* tests.

Compared with control cells, *Ndufs4^fky/fky^* primary MEFs generated increased amounts of O_2_^•−^ at rest on glucose medium (Figure 5A; 150% of +/+), but not on galactose medium (Figure 5B). In comparison, the rates of O_2_^•−^ production at rest in *Ndufs4^fky/fky^* primary astrocytes cultured on either glucose (Figure 5D) or galactose (Figure 5E) medium were indistinguishable from controls. Rotenone treatment stimulated O_2_^•−^ in all cell types, independent of genotype or culture conditions (Figures 5A, 5B, 5D and 5E). However, the rotenone dependent increase in O_2_^•−^ production was less for both *Ndufs4^fky/fky^* primary MEFs (Figure 5C) and astrocytes (Figure 5F) compared with +/+, regardless of the culture media.

In contrast, the rates of H_2_O_2_ production were equivalent to controls in isolated mitochondria from *Ndufs4^fky/fky^* MEFs (Figure 5G) and astrocytes (Figure 5H) with both CI- (glutamate+malate) and CII-dependent substrates (succinate). As well, the rotenone induced increase in H_2_O_2_ production linked to CI was equivalent in mitochondria from both +/+ and *Ndufs4^fky/fky^* primary MEFs and astrocytes (Figures 5G and 5H). Notably though, the CII-dependent rate of H_2_O_2_ production in both MEF and astrocyte mitochondria was lower when incubated with the CI-inhibitor rotenone than without. Moreover, although not significant, this trend appears to be more pronounced in both *Ndufs4^fky/fky^* MEFs and astrocytes compared to controls. This further suggests the occurrence of RET in our primary cells, and that the process is attenuated in *Ndufs4^fky/fky^* cells.

### *Ndufs4^fky/fky^* primary MEFs are more susceptible to entering cell death on galactose

**Figure 6:**
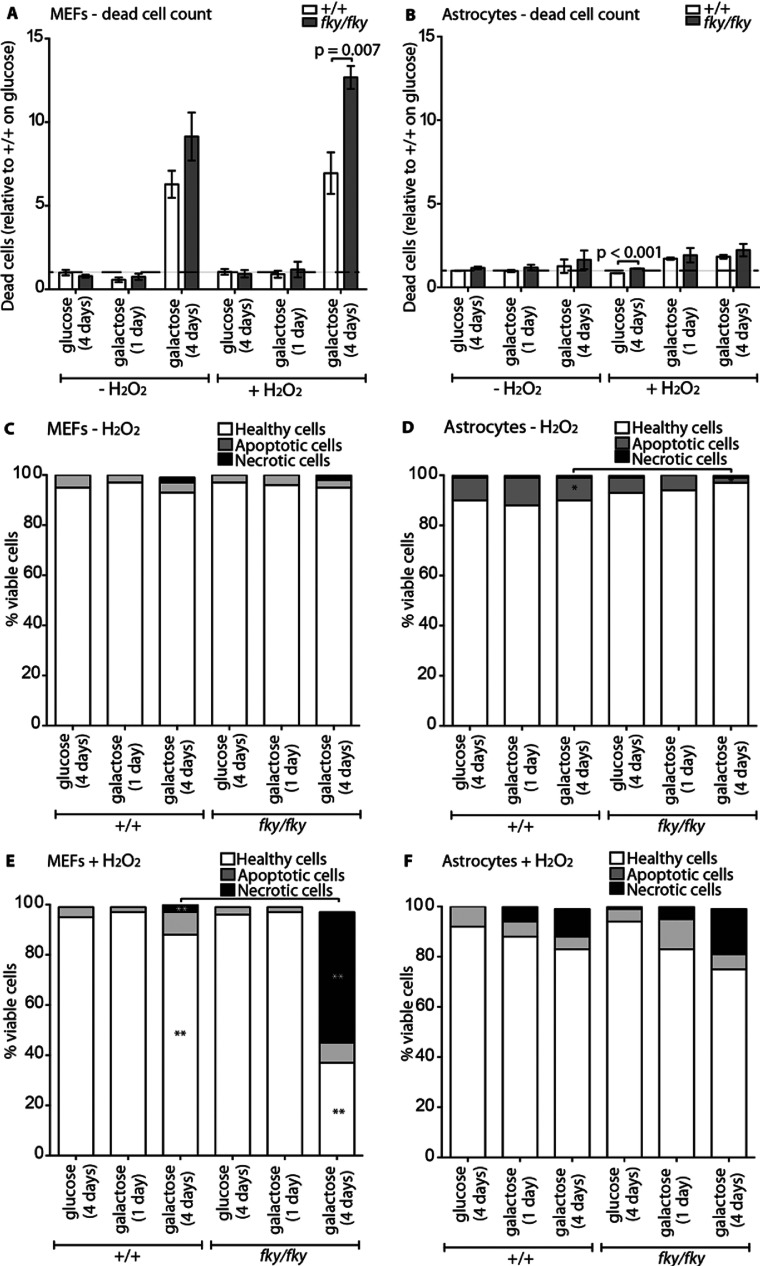
Cell death in *Ndufs4^fky/fky^* cell lines The propensity of primary MEFs and astrocytes from +/+ and *Ndufs4^fky/fky^* mice to enter cell death was measured by flow cytometry. The ratio of dead cells relative to +/+ (on glucose medium) for primary MEFs is presented in (**A**) and astrocytes (**B**), either cultured on glucose medium for 1 day or galactose medium for 1 day or 4 days, with or without acute H_2_O_2_ treatment for 6 h prior to analysis. The percentage of viable single-celled primary MEFs is presented in (**C**, **E**) and astrocytes (**D**, **F**), either cultured on glucose for 1 day or galactose for 1 day or 4 days, with (**E**, **F**) or without (**C**, **D**) acute H_2_O_2_ treatment for 6 h prior to analysis. Error bars=S.E.M., *n*≥4, *P* values generated from two-tailed unpaired *t* tests correcting for multiple comparisons using the Holm–Sidak method (GraphPad, PRISM V6.0b).*=*P*≤0.050, **=*P*≤0.005. PI, propidium iodide.

Our results suggest that alterations to mitochondrial function are cell type specific, whereby under standard culture conditions on glucose medium, *Ndufs4^fky/fky^* primary MEFs manifest a reduced ΔΨ_m_ and increased ROS production, while the astrocytes and neurons exhibit a reduced CI-dependent ATP synthesis capacity. Moreover, *Ndufs4^fky/fky^* primary MEFs, but not astrocytes, appeared on average to be more sensitive to maintenance on galactose, as evidenced by a further depolarization of the ΔΨ_m_ and a corresponding drop in ATP synthesis. All these processes have the potential to affect initiation of cell death [41]. Accordingly, we examined cell death in *Ndufs4^fky/fky^* primary cells. These investigations revealed that *Ndufs4^fky/fky^* primary MEFs (Figures 6A, 6C and 6E), but not astrocytes (Figures 6B, 6D and 6F), were more sensitive to cell death (see Supplementary Table S1 for statistics on analyses of viable cells). However, this increased sensitivity to cell death was only observed in *Ndufs4^fky/fky^* primary MEFs when maintained on galactose for an extended period of time, followed by the application of the acute stressor H_2_O_2_. Not only was there an increased percentage of dead cells at the time of analysis (Figure 6A; 1.8 times +/+), but an increased percentage of viable cells that were entering into what appeared to be necrotic cell death (Figure 6E; 13 times +/+). In contrast to *Ndufs4^fky/fky^* primary MEFs, *Ndufs4^fky/fky^* astrocytes (Figures 6B, 6D and 6F) displayed no marked change in sensitivity to cell death compared to +/+ under all culture conditions tested.

## DISCUSSION

It is generally accepted that OXPHOS mutations likely cause cell-specific effects, due to the differential energy requirements and metabolic pathways utilized by various cell and tissue types [9,42]. Here, we sought to characterize this further by analysing mitochondrial function in neurological and non-neurological primary cell types from the *Ndufs4^fky/fky^* mouse model mimicking aspects of LS [12]. In conjunction, we used culture conditions (galactose) that forced *Ndufs4^fky/fky^* primary MEFs and astrocytes to rely less on glycolytic ATP generation and more on OXPHOS linked ATP production to emphasize any underlying mitochondrial defects. Astrocytes in particular have been shown to up-regulate glycolysis in response to respiratory inhibition [43,44]. This study significantly makes direct comparisons between primary cells derived from the same animal model of OXPHOS deficiency, the *Ndufs4^fky/fky^* mouse. As well, it encompasses one of the first comprehensive analyses of astrocytes from a mammalian model of mitochondrial CI deficiency.

*Ndufs4^fky/fky^* MEFs were chosen as a control non-neurological cell type, since OXPHOS-deficient patient fibroblasts are the source of much of our current knowledge regarding the cellular biochemistry of mitochondrial diseases [45]. The biochemical profile under standard culture conditions of *Ndufs4^fky/fky^* primary MEFs described in the present study closely matches studies conducted in NDUFS4-deficient human and mouse fibroblast model systems. Notably, CI activity is reduced, CI assembly is impaired, the ΔΨ_m_ is slightly depolarized, and ROS production is typically increased [15–17,46,47]. These data suggest that disease pathogenesis could partly result from a compromised ΔΨ_m_, which is essential for maintaining basic cellular functions such as ATP synthesis, mitochondrial protein import, cellular Ca^2+^ homoeostasis, and ultimately cell survival. Furthermore, ROS are also implicated in disease progression, causing oxidative damage to cellular structures. However, since LS is primarily a neurological disorder, fibroblasts are not well suited for the study of mechanisms contributing to the neuropathology.

We find then that the biochemical profile of *Ndufs4^fky/fky^* primary MEFs is divergent from that of the neurologically relevant astrocytes and neurons. That is, despite both neurological and non-neurological *Ndufs4^fky/fky^* primary cell types manifesting reduced CI activity and disrupted CI formation, the effect on mitochondrial function differs. In particular, *Ndufs4^fky/fky^* primary astrocytes and neurons have a remarkably normal ΔΨ_m_ under standard culture conditions. Nonetheless, our data indicate that the CI-dependent ATP synthesis rates in *Ndufs4^fky/fky^* primary astrocytes and neurons is reduced, paralleling previous studies in brain from the *Ndufs4^fky/fky^* mouse also reporting reduced CI-dependent ATP synthesis (65% of +/+) [12]. This represents the opposite trend to the *Ndufs4^fky/fky^* primary MEFs, perhaps alluding to the maintenance of the ΔΨ_m_ as an adaptive response by the *Ndufs4^fky/fky^* neurological cell types to protect against cell death. Such a response might result from modulation of CV activity, perhaps via altering levels of the IF_1_ regulatory protein [48] or reverse action of the complex [49,50]. It should be noted though that we have reported maximal rates of ATP synthesis, while the ΔΨ_m_ was monitored under steady state conditions. Also, neurons themselves may be more sensitive to the disruption of CI activity than other cell types as indicated by our results that neurons have a reduced CI capacity relative to mitochondrial volume and/or number in comparison to astrocytes and MEFs.

Moreover, while increased ROS production was a feature in NDUFS4 deficient patient and mouse fibroblasts [17,46], there was little evidence of this in our *Ndufs4^fky/fky^* primary astrocytes, nor the previously described NDUFS4 deficient mesencephalic neurons [20,21]. Previous cellular studies have indicated correlations between the degree of CI inhibition and the level of ROS production [18,51]. In contrast, we observed variations in ROS production between different cell types from the same mouse model with the same level of CI inhibition. This variation could partly result from differing levels of cellular anti-oxidants, such as the decreased glutathione observed in neurons versus astrocytes [52,53]. Again, this emphasizes the pathogenic relevance of investigating these parameters in appropriate cells. As well, it should be noted that both *Ndufs4^fky/fky^* primary MEFs and astrocytes showed a smaller increase in ROS generation after addition of rotenone. This may reflect an adaptation to the loss of NDUFS4 and the unstable CI observed *in vitro* in these cells, such that the basal rate of ROS generation in CI-deficient cells is closer to the maximal rate than in +/+ cells.

Nonetheless, oxidative damage has been observed in the brain of *Ndufs4* KO mice [13]. Although oxidative damage may be a major contributing factor to disease, the source of ROS is likely to be external from the affected cell types. Cavanagh and Harding [7] suggested that oxidative damage to neurological tissue in LS patients is mediated by the effect of pH changes to the neurovasculature (hyperlacticacidaemia). Moreover, these pH changes could be more pronounced at a local level in the brain due to its high rate of metabolic activity [7,54]. Combined with the highly oxygenated environment of the mammalian brain, conditions may be conducive to the catalysis of ROS formation, thus mediating oxidative damage in affected brain regions [7]. Alternatively, oxidative damage in the brain may result from gliosis, an infiltration of activated glial cells, including astrocytes. Gliosis is a feature of LS [7,8], and was also observed in *Ndufs4* KO mice [13,14]. These activated glial cells may release pro-inflammatory cytokines, further recruiting immune cells to the affected brain region, producing a burst of ROS and driving oxidative damage [7,13,14,55]. Furthermore, aberrant activation of astrocytes could cause tissue damage by promoting inflammation, contributing to scarring and preventing axonal regrowth from neurons [55,56]. Indeed with few exceptions, there is little correlation between the ROS production and oxidative damage observed in neurologically relevant models of mitochondrial disease [9].

This study also finds evidence to suggest that electrons from ubiquinol (QH_2_) generated by succinate oxidation at CII can be transferred in both the forward direction to CIII and the reverse direction to CI (RET) [38,57]. This was indicated by both the increased efficiency of the CII-dependent rate of ATP synthesis and the higher rate of H_2_O_2_ production, when measured in the presence of the CI inhibitor rotenone. Notably, the trend appears to be more pronounced in neurons than in primary MEFs or astrocytes, and is attenuated in *Ndufs4^fky/fky^* primary cells compared with controls. The physiological relevance of this phenomenon to disease is unclear, although RET-induced changes in redox balance or generation of ROS may influence cell signalling. It is therefore of interest that neurons have higher rates of RET than other cells. Nonetheless, as these experiments measured maximal rates of activity, they likely do not reflect the *in vivo* activity of the system. Moreover, the measurements describing RET were performed in the absence of CI-dependent substrates, unlike in the *in vivo* environment where CI substrates may influence forward electron transport through CI.

When grown with galactose instead of glucose, to force OXPHOS dependent respiration, *Ndufs4^fky/fky^* primary MEFs again phenotypically deviated from astrocytes. While *Ndufs4^fky/fky^* primary MEFs responded by further depolarizing their ΔΨ_m_ with a corresponding reduction in CI-dependent ATP synthesis capacity, *Ndufs4^fky/fky^* primary astrocytes responded no differently than when maintained under standard culture conditions. Moreover, *Ndufs4^fky/fky^* primary MEFs on galactose have an increased propensity for cell death, while *Ndufs4^fky/fky^* primary astrocytes do not. This suggests that *Ndufs4^fky/fky^* primary MEFs have insufficient reserve OXPHOS capacity to adapt to maintenance on galactose. In contrast, *Ndufs4^fky/fky^* astrocytes may have a larger spare OXPHOS capacity, or the ability to reduce their metabolic demand more readily, thus preferentially maintaining their ΔΨ_m_. This difference however was only significant with the acute application of H_2_O_2_, again perhaps indicative of underlying differences in the antioxidant defence systems in these two cell types that were not investigated in the present study.

*Ndufs4^fky/fky^* primary neurological cell types may preferentially maintain some mitochondrial processes over others, such as the ΔΨ_m_ over ATP synthesis, to avoid sensitization to cell death. This would be consistent with the phenomenon described in LS patients known as relative neuronal sparing, characterized by the preservation of neurons in localized regions of cell death [7,8]. Likewise, it was observed in CI-deficient *mt-Nd5/6* transgenic cybrid-derived neurons and astrocytes that the ΔΨ_m_ was elevated by up to 40% under resting conditions [53], yet collapsed with application of the CV inhibitor oligomycin. Furthermore, neurons are known to possess other mechanisms to evade cell death, such as their reduced sensitivity to cytochrome *c*-mediated apoptosis [58].

Considering that the CI-dependent ATP synthesis capacity of *Ndufs4^fky/fky^* astrocytes and neurons is greatly reduced, energy-dependent processes in these cells, such as the capacity to generate action potentials, may be compromised. Likewise, this may impact the astrocytes’ ability to recycle glutamine and potassium released as neurotransmitters by neurons, maintain the blood–brain barrier and propagate action potentials [23,24,56,59]. The effect of the CI deficiency on the capacity of astrocytes to recycle neurotransmitters released by neurons is of particular importance, given that this process alone likely accounts for a large proportion of the brain's energy budget, and may be hypersensitive to an ATP deficiency [54].

Collectively, we show that *Ndufs4^fky/fky^* primary MEFs respond differently to the CI deficiency compared with the astrocytes and neurons, which may reflect that the tissue from which the MEFs are derived is less metabolically active than brain. Furthermore, measurements obtained from the primary astrocytes and neurons were conducted at rest and it remains to be determined how robust mitochondrial function is in stimulated cells. Ultimately, these results highlight the need to study disease mechanisms in relevant cells and tissues, since studies in less relevant samples, such as fibroblasts, may be misleading.

## Online data

Supplementary data

## References

[1] Skladal D., Halliday J., Thorburn D. R. (2003). Minimum birth prevalence of mitochondrial respiratory chain disorders in children. Brain.

[2] Munnich A., Rustin P. (2001). Clinical spectrum and diagnosis of mitochondrial disorders. Am. J. Med. Genet..

[3] Pfeffer G., Chinnery P. F. (2013). Diagnosis and treatment of mitochondrial myopathies. Ann. Med..

[4] Koopman W. J. H., Distelmaier F., Smeitink J. A. M., Willems P. H. G. M. (2013). OXPHOS mutations and neurodegeneration. EMBO J..

[5] DiMauro S., Schon E. A. (2008). Mitochondrial disorders in the nervous system. Annu. Rev. Neurosci..

[6] Baertling F., Rodenburg R. J., Schaper J., Smeitink J. A., Koopman W. J. H., Mayatepek E., Morava E., Distelmaier F. (2014). A guide to diagnosis and treatment of Leigh syndrome. J. Neurol. Neurosurg. Psychiatry.

[7] Cavanagh J. B., Harding B. N. (1994). Pathogenic factors underlying the lesions in Leigh's disease. Tissue responses to cellular energy deprivation and their clinico-pathological consequences. Brain.

[8] Rahman S., Blok R. B., Dahl H. H., Danks D. M., Kirby D. M., Chow C. W., Christodoulou J., Thorburn D. R. (1996). Leigh syndrome: clinical features and biochemical and DNA abnormalities. Ann. Neurol..

[9] Bird M. J., Thorburn D. R., Frazier A. E. (2014). Modelling biochemical features of mitochondrial neuropathology. Biochim. Biophys. Acta.

[10] Wallace D. C., Fan W. (2009). The pathophysiology of mitochondrial disease as modeled in the mouse. Genes Dev..

[11] Kruse S. E., Watt W. C., Marcinek D. J., Kapur R. P., Schenkman K. A., Palmiter R. D. (2008). Mice with mitochondrial complex I deficiency develop a fatal encephalomyopathy. Cell Metab..

[12] Leong D. W., Komen J. C., Hewitt C. A., Arnaud E., McKenzie M., Phipson B., Bahlo M., Laskowski A., Kinkel S. A., Davey G. M. (2012). Proteomic and metabolomic analyses of mitochondrial complex I-deficient mouse model generated by spontaneous B2 short interspersed nuclear element (SINE) insertion into NADH dehydrogenase (ubiquinone) Fe–S protein 4 (Ndufs4) gene. J. Biol. Chem..

[13] Quintana A., Kruse S. E., Kapur R. P., Sanz E., Palmiter R. D. (2010). Complex I deficiency due to loss of Ndufs4 in the brain results in progressive encephalopathy resembling Leigh syndrome. Proc. Natl. Acad. Sci. U. S. A..

[14] Quintana A., Zanella S., Koch H., Kruse S. E., Lee D., Ramirez J. M., Palmiter R. D. (2012). Fatal breathing dysfunction in a mouse model of Leigh syndrome. J. Clin. Invest..

[15] Assouline Z., Jambou M., Rio M., Bole-Feysot C., de Lonlay P., Barnerias C., Desguerre I., Bonnemains C., Guillermet C., Steffann J. (2012). A constant and similar assembly defect of mitochondrial respiratory chain complex I allows rapid identification of NDUFS4 mutations in patients with Leigh syndrome. Biochim. Biophys. Acta.

[16] Valsecchi F., Monge C., Forkink M., de Groof A. J. C., Benard G., Rossignol R., Swarts H. G., van Emst-de Vries S. E., Rodenburg R. J., Calvaruso M. A. (2012). Metabolic consequences of NDUFS4 gene deletion in immortalized mouse embryonic fibroblasts. Biochim. Biophys. Acta.

[17] Valsecchi F., Grefte S., Roestenberg P., Joosten-Wagenaars J., Smeitink J. A. M., Willems P. H. G. M., Koopman W. J. H. (2013). Primary fibroblasts of NDUFS4^−/−^ mice display increased ROS levels and aberrant mitochondrial morphology. Mitochondrion.

[18] Verkaart S., Koopman W. J. H., van Emst-de Vries S. E., Nijtmans L. G. J., van den Heuvel L. W. P. J., Smeitink J. A. M., Willems P. H. G. M. (2007). Superoxide production is inversely related to complex I activity in inherited complex I deficiency. Biochim. Biophys. Acta.

[19] Verkaart S., Koopman W. J. H., Cheek J., van Emst-de Vries S. E., van den Heuvel L. W. P. J., Smeitink J. A. M., Willems P. H. G. M. (2007). Mitochondrial and cytosolic thiol redox state are not detectably altered in isolated human NADH: ubiquinone oxidoreductase deficiency. Biochim. Biophys. Acta.

[20] Choi W.-S., Kruse S. E., Palmiter R. D., Xia Z. (2008). Mitochondrial complex I inhibition is not required for dopaminergic neuron death induced by rotenone, MPP+, or paraquat. Proc. Natl. Acad. Sci. U. S. A..

[21] Choi W.-S., Palmiter R. D., Xia Z. (2011). Loss of mitochondrial complex I activity potentiates dopamine neuron death induced by microtubule dysfunction in a Parkinson's disease model. J. Cell Biol..

[22] Freeman M. R. (2010). Specification and morphogenesis of astrocytes. Science.

[23] Barres B. A. (2008). The mystery and magic of glia: a perspective on their roles in health and disease. Neuron.

[24] Dienel G. A. (2013). Astrocytic energetics during excitatory neurotransmission: What are contributions of glutamate oxidation and glycolysis?. Neurochem. Int..

[25] Du F., Qian Z. M., Zhu L., Wu X. M., Qian C., Chan R., Ke Y. (2010). Purity, cell viability, expression of GFAP and bystin in astrocytes cultured by different procedures. J. Cell. Biochem..

[26] Meberg P. J., Miller M. W. (2003). Culturing hippocampal and cortical neurons. Methods Cell Biol..

[27] Trounce I. A., Schmiedel J., Yen H. C., Hosseini S., Brown M. D., Olson J. J., Wallace D. C. (2000). Cloning of neuronal mtDNA variants in cultured cells by synaptosome fusion with mtDNA-less cells. Nucleic Acids Res..

[28] McKenzie M., Lazarou M., Thorburn D. R., Ryan M. T. (2006). Mitochondrial respiratory chain supercomplexes are destabilized in Barth Syndrome patients. J. Mol. Biol..

[29] Frazier A. E., Thorburn D. R. (2012). Biochemical analyses of the electron transport chain complexes by spectrophotometry. Methods Mol. Biol..

[30] McKenzie M., Lazarou M., Thorburn D. R., Ryan M. T. (2007). Analysis of mitochondrial subunit assembly into respiratory chain complexes using Blue Native polyacrylamide gel electrophoresis. Anal. Biochem..

[31] Dunning C. J., McKenzie M., Sugiana C., Lazarou M., Silke J., Connelly A., Fletcher J. M., Kirby D. M., Thorburn D. R., Ryan M. T. (2007). Human CIA30 is involved in the early assembly of mitochondrial complex I and mutations in its gene cause disease. EMBO J..

[32] Wanders R. J., Ruiter J. P., Wijburg F. A. (1993). Studies on mitochondrial oxidative phosphorylation in permeabilized human skin fibroblasts: application to mitochondrial encephalomyopathies. Biochim. Biophys. Acta.

[33] Morten K. J., Ackrell B. A., Melov S. (2006). Mitochondrial reactive oxygen species in mice lacking superoxide dismutase 2: attenuation via antioxidant treatment. J. Biol. Chem..

[34] Calvaruso M. A., Willems P., van den Brand M., Valsecchi F., Kruse S., Palmiter R., Smeitink J., Nijtmans L. (2012). Mitochondrial complex III stabilizes complex I in the absence of NDUFS4 to provide partial activity. Hum. Mol. Genet..

[35] Appleby R. D., Porteous W. K., Hughes G., James A. M., Shannon D., Wei Y.-H., Murphy M. P. (1999). Quantitation and origin of the mitochondrial membrane potential in human cells lacking mitochondrial DNA. Eur. J. Biochem..

[36] Buchet K., Godinot C. (1998). Functional F1-ATPase essential in maintaining growth and membrane potential of human mitochondrial DNA-depleted ρ° cells. J. Biol. Chem..

[37] Boyer P. D. (1997). The ATP synthase–a splendid molecular machine. Annu. Rev. Biochem..

[38] Dröse S., Brandt U. (2012). Molecular mechanisms of superoxide production by the mitochondrial respiratory chain. Adv. Exp. Med. Biol..

[39] Balaban R. S., Nemoto S., Finkel T. (2005). Mitochondria, oxidants, and aging. Cell.

[40] Venditti P., Di Stefano L., Di Meo S. (2013). Mitochondrial metabolism of reactive oxygen species. Mitochondrion.

[41] Galluzzi L., Kepp O., Kroemer G. (2012). Mitochondria: master regulators of danger signalling. Nat. Rev. Mol. Cell Biol..

[42] Lombès A., Auré K., Bellanné-Chantelot C., Gilleron M., Jardel C. (2014). Unsolved issues related to human mitochondrial diseases. Biochimie.

[43] Bolaños J. P., Almeida A., Moncada S. (2010). Glycolysis: a bioenergetic or a survival pathway?. Trends Biochem. Sci..

[44] Bouzier-Sore A. K., Pellerin L. (2013). Unraveling the complex metabolic nature of astrocytes. Front. Cell. Neurosci..

[45] Saada A. (2014). Mitochondria: mitochondrial OXPHOS (dys) function *ex vivo*–The use of primary fibroblasts. Int. J. Biochem. Cell Biol..

[46] Distelmaier F., Koopman W. J. H., Van Den Heuvel L. P., Rodenburg R. J., Mayatepek E., Willems P. H. G. M., Smeitink J. A. M. (2009). Mitochondrial complex I deficiency: from organelle dysfunction to clinical disease. Brain.

[47] Lazarou M., McKenzie M., Ohtake A., Thorburn D. R., Ryan M. T. (2007). Analysis of the assembly profiles for mitochondrial- and nuclear-DNA-encoded subunits into complex I. Mol. Cell. Biol..

[48] Campanella M., Casswell E., Chong S., Farah Z., Wieckowski M. R., Abramov A. Y., Tinker A., Duchen M. R. (2008). Regulation of mitochondrial structure and function by the F1Fo-ATPase inhibitor protein, IF1. Cell Metab..

[49] McKenzie M., Liolitsa D., Akinshina N., Campanella M., Sisodiya S., Hargreaves I., Nirmalananthan N., Sweeney M. G., Abou-Sleiman P. M., Wood N. W. (2007). Mitochondrial ND5 gene variation associated with encephalomyopathy and mitochondrial ATP consumption. J. Biol. Chem..

[50] Chinopoulos C., Adam-Vizi V. (2010). Mitochondria as ATP consumers in cellular pathology. Biochim. Biophys. Acta.

[51] Barrientos A., Moraes C. T. (1999). Titrating the effects of mitochondrial complex I impairment in the cell physiology. J. Biol. Chem..

[52] Dringen R., Hirrlinger J. (2003). Glutathione pathways in the brain. Biol. Chem..

[53] Abramov A. Y., Smulders-Srinivasan T. K., Kirby D. M., Acin-Perez R., Enriquez J. A., Lightowlers R. N., Duchen M. R., Turnbull D. M. (2010). Mechanism of neurodegeneration of neurons with mitochondrial DNA mutations. Brain.

[54] Mergenthaler P., Lindauer U., Dienel G. A., Meisel A. (2013). Sugar for the brain: the role of glucose in physiological and pathological brain function. Trends Neurosci..

[55] Singh S., Swarnkar S., Goswami P., Nath C. (2011). Astrocytes and microglia: responses to neuropathological conditions. Int. J. Neurosci..

[56] Sofroniew M. V. (2005). Reactive astrocytes in neural repair and protection. Neuroscientist.

[57] Pryde K. R., Hirst J. (2011). Superoxide is produced by the reduced flavin in mitochondrial complex I: a single, unified mechanism that applies during both forward and reverse electron transfer. J. Biol. Chem..

[58] Vaughn A. E., Deshmukh M. (2008). Glucose metabolism inhibits apoptosis in neurons and cancer cells by redox inactivation of cytochrome *c*. Nat. Cell Biol..

[59] Walz W. (2000). Role of astrocytes in the clearance of excess extracellular potassium. Neurochem. Int..

