# Supplementary material for: Neuronal and astrocyte dysfunction diverges from embryonic fibroblasts in the Ndufs4fky/fky mouse
Source: Biosci Rep. 2014 Nov 21;34(6):e00151. doi: 10.1042/BSR20140151 (PMC4240023; doi:10.1042/BSR20140151)
Supplement: Supplementary data [file bsr034e151ntsadd.pdf]

Supplemental methods

Immunocytochemistry

The purity of primary astrocyte and neuronal cell cultures was determined by immunocytochemistry (Figure S1A, B) according to standard procedures (Harlow and Lane, 1999). Briefly, cells cultured on glass coverslips were fixed in 4% paraformaldehyde and permeabilised with 0.2% Triton X-100. Primary antibody was applied [anti-glial fibrillary acidic protein (Millipore, MAB360), or anti-NeuN (Millipore, MAB377)], followed by secondary anti-mouse Alexa Fluor 578 (Molecular Probes). Slides were then stained with 0.5µg/ml Hoechst-33258 and mounted in VECTASHIELD (Vector Laboratories). Cells were visualised with a Zeiss Axio Imager M1 fluorescence microscope at 20 X magnification. The percentage of primary astrocytes (Figure S1A) was observed to be 89% ± 4.0 (SEM) for +/+ and 79% ± 4.9 for *fky/fky* preparations (not statistically significant). The percentage of primary neurons (Figure S1B) was observed to be 86% ± 8.0 for +/+ and 93% ± 1.7 for *fky/fky* preparations (not statistically significant).

Reference

Harlow, E., and Lane, D. (1999). Using Antibodies: A Laboratory Manual, New York: Cold Spring Harbor Laboratory Press.

Table S1 Cell death statistics in +/+ and *Ndufs4*$^{fky/fky}$ cell lines.

Cells (Treatment)	Genotype	Medium	Healthy cells[a]	Apoptotic cells[a]	Necrotic cells[a]
MEFs (– H_2O_2)	+/+	DMEM (glucose)	95 ± 1.5 (3)	4.7 ± 1.5 (3)	0.0 ± 0.0 (3)
		DMEM (galactose –24 hrs)	97 ± 0.6 (3)	3.0 ± 0.6 (3)	0.0 ± 0.0 (3)
		DMEM (galactose –96 hrs)	93 ± 0.6 (3)	4.3 ± 0.7 (3)	1.7 ± 0.3 (3)
	fky/fky	**DMEM (glucose)**	**97 ± 0.9 (3)**	**3.0 ± 0.7 (3)**	**0.0 ± 0.0 (3)**
		**DMEM (galactose –24 hrs)**	**96 ± 0.9 (3)**	**3.5 ± 0.9 (3)**	**0.0 ± 0.0 (3)**
		**DMEM (galactose –96 hrs)**	**95 ± 0.6 (3)**	**3.0 ± 0.3 (3)**	**3.7 ± 0.0 (3)**
MEFs (+ H_2O_2)	+/+	DMEM (glucose)	95 ± 0.9 (3)	4.3 ± 0.9 (3)	0.0 ± 0.0 (3)
		DMEM (galactose –24 hrs)	97 ± 0.9 (3)	2.3 ± 0.9 (3)	0.0 ± 0.0 (3)
		DMEM (galactose –96 hrs)	88 ± 2.7 (3)*	8.7 ± 2.3 (3)	3.7 ± 0.3 (3)‡
	fky/fky	**DMEM (glucose)**	**96 ± 0.9 (3)**	**3.3 ± 0.9 (3)**	**0.0 ± 0.0 (3)**
		**DMEM (galactose –24 hrs)**	**97 ± 0.6 (3)**	**1.8 ± 0.6 (3)**	**0.3 ± 0.3 (3)**
		**DMEM (galactose –96 hrs)**	**37 ± 6.2 (3)***	**8.0 ± 0.7 (3)**	**52 ± 7.1 (3)‡**
Astrocytes (– H_2O_2)	+/+	DMEM (glucose)	90 ± 3.8 (3)	9.0 ± 3.8 (3)	0.7 ± 0.3 (3)
		DMEM (galactose –24 hrs)	88 ± 2.0 (3)	11 ± 2.3 (3)	0.7 ± 0.3 (3)
		DMEM (galactose –96 hrs)	90 ± 1.8 (3)	9.3 ± 1.7 (3)†	1.0 ± 0.0 (3)
	fky/fky	**DMEM (glucose)**	**93 ± 1.0 (2)**	**5.5 ± 0.5 (2)**	**0.5 ± 0.5 (2)**
		**DMEM (galactose –24 hrs)**	**94 ± 0.5 (2)**	**5.5 ± 0.5 (2)**	**1.0 ± 0.0 (2)**
		**DMEM (galactose –96 hrs)**	**97 ± 1.0 (2)**	**2.0 ± 1.0 (2)†**	**1.0 ± 0.0 (2)**
Astrocytes (+ H_2O_2)	+/+	DMEM (glucose)	92 ± 2.2 (3)	7.7 ± 2.4 (3)	0.3 ± 0.3 (3)
		DMEM (galactose –24 hrs)	88 ± 1.8 (3)	6.0 ± 0.6 (3)	5.7 ± 2.0 (3)
		DMEM (galactose –96 hrs)	83 ± 8.9 (3)	5.3 ± 0.7 (3)	11 ± 9.8 (3)
	fky/fky	**DMEM (glucose)**	**94 ± 1.0 (2)**	**5.0 ± 1.0 (2)**	**0.5 ± 0.5 (2)**
		**DMEM (galactose –24 hrs)**	**83 ± 2.0 (2)**	**12 ± 0.5 (2)**	**5.0 ± 2.0 (2)**
		**DMEM (galactose –96 hrs)**	**75 ± 14 (2)**	**6.0 ± 0.0 (2)**	**18 ± 13 (2)**

[a]Percentage viable cells gated by flow cytometry using the FITC-A and PI-488-695/40-A lasers ± SEM (n). *,†,‡Statistical significance between +/+ and *fky/fky* at the $p \leq 0.050$ level as measured by two-tailed unpaired t-tests correcting for multiple comparisons using the Holm-Sidak method (GraphPad, PRISM V6.0b).

Supplemental Figures

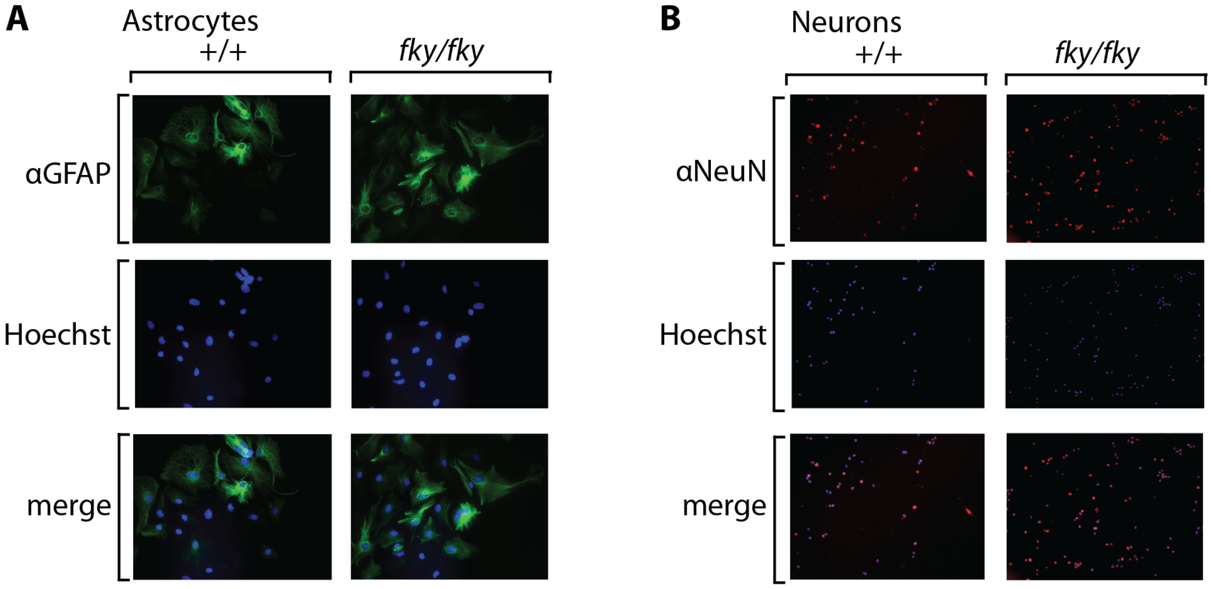

Figure S1. Cell culture purity in *Ndufs4*$^{fky/fky}$ cell lines. Immunocytochemistry was performed on primary astrocyte and neuronal cell cultures from +/+ and *Ndufs4*$^{fky/fky}$ mice using (A) anti-glial fibrillary acidic protein (GFAP, green), and (B) anti-NeuN (red), respectively. Nuclei were counterstained with Hoechst (blue).

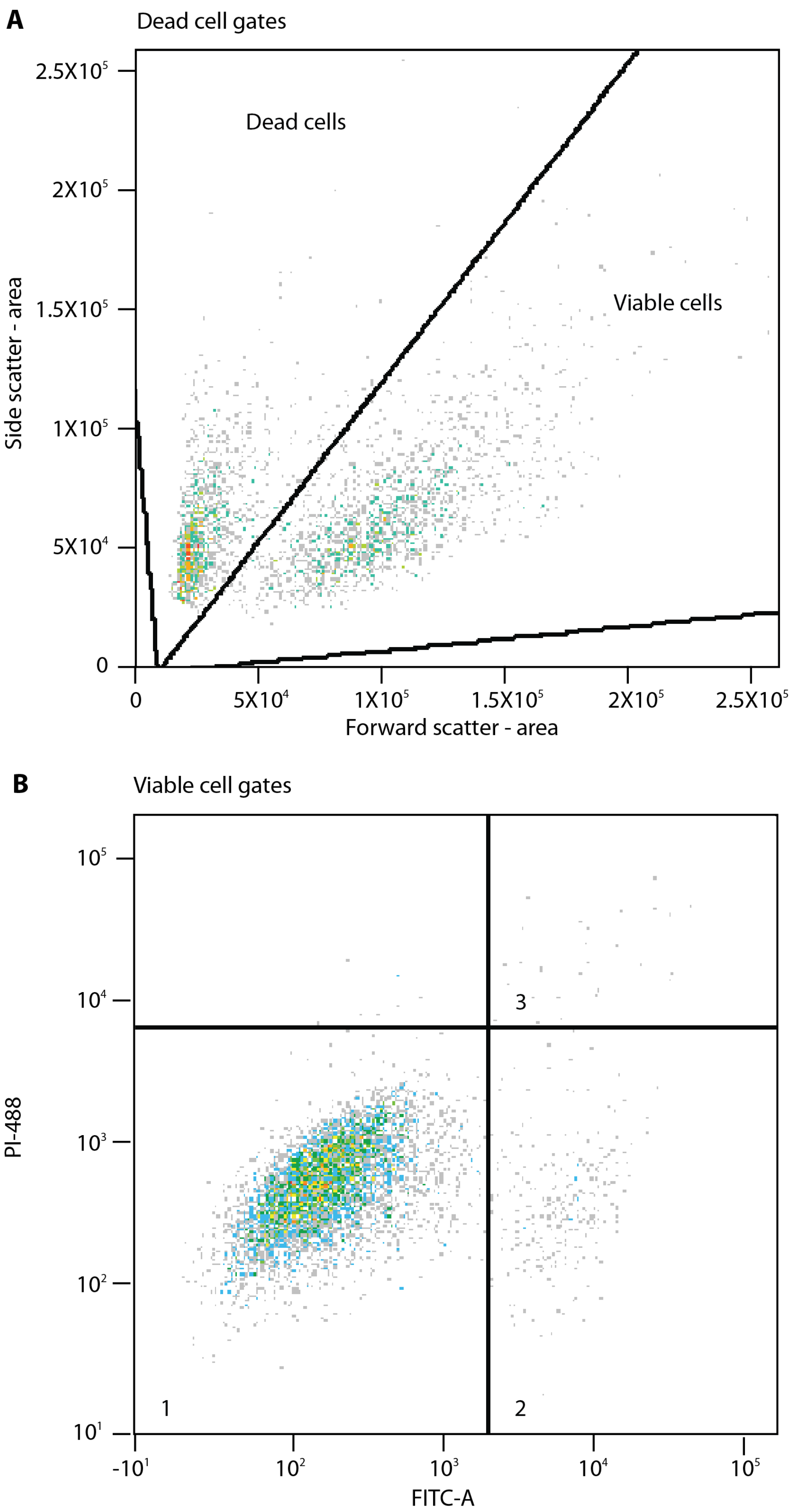
A
Dead cell gates
Dead cells
Viable cells
Side scatter - area
Forward scatter - area
2.5X10^5
2X10^5
1.5X10^5
1X10^5
5X10^4
0
B
Viable cell gates
PI-488
FITC-A
10^5
10^4
10^3
10^2
10^1
-10^1
1
2
3

Figure S2. Gating strategy for flow cytometry. Trypsinized cells were loaded with annexin V-FITC and PI. The percentage of dead cells was monitored by forward scatter-area versus side scatter-area as shown in the representative plot (A). Viable cells were further gated for annexin V-FITC (FITC-A laser) and PI (PI-488 laser) positive staining as shown in the representative plot (B) delineated quadrant 1 (healthy cells, double negative), quadrant 2 (apoptotic cells, annexin V-FITC positive) or quadrant 3 (late stage apoptotic or necrotic, annexin V-FITC and PI double positive). Abbreviations: PI, propidium iodide.

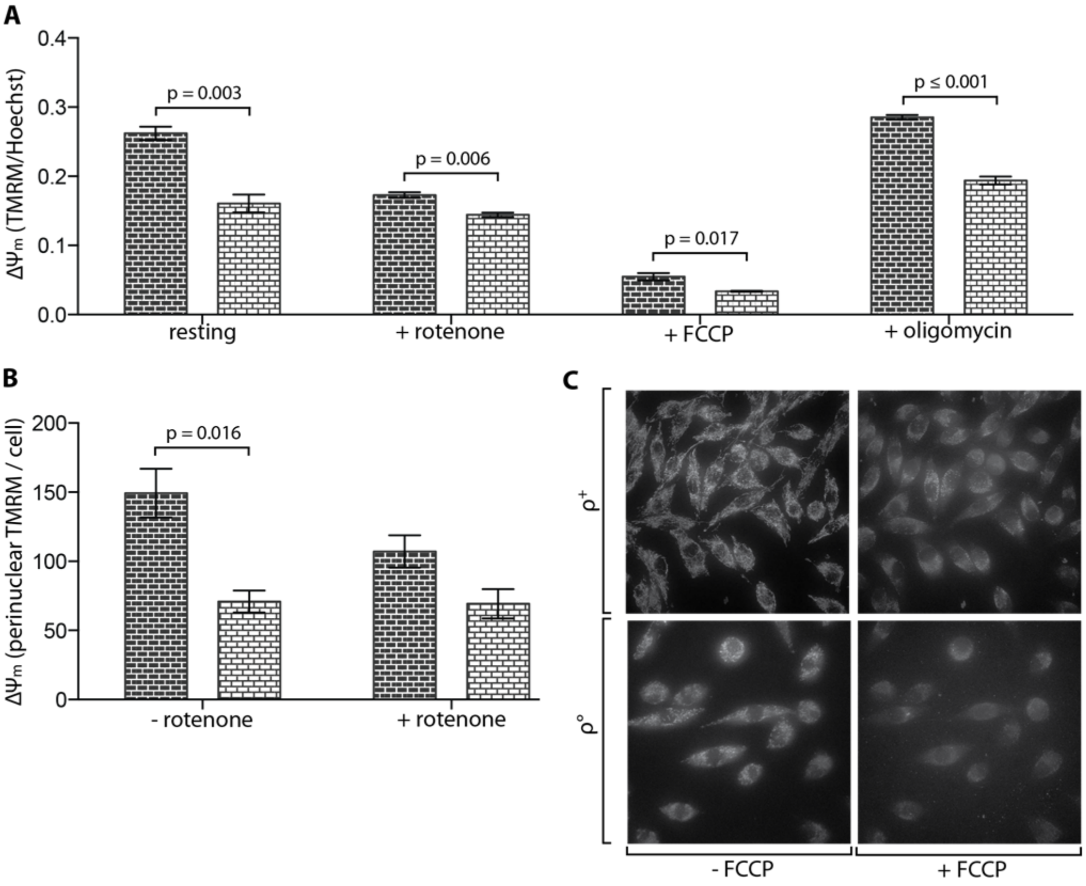

Figure S3. Analysis of the $\Delta\Psi_m$ in ρ° cells. The $\Delta\Psi_m$ was measured by the accumulation of TMRM in mitochondria and made relative to cell number in mtDNA deficient mouse ρ° (white bricks) cells or their control (ρ^+, black bricks) (A). The $\Delta\Psi_m$ was reduced with the CI inhibitor rotenone or the protonophore FCCP, or modulated by the CV inhibitor oligomycin, as indicated. The $\Delta\Psi_m$ was alternatively assessed by microscopy in mouse ρ cells (B) pre- and post-incubation with rotenone, by measuring the accumulation of the cationic dye TMRM in the mitochondrial rich perinuclear region. Greater than 60 cells per dish were analysed using ≥ 3 dishes. Representative images are shown in (C), pre- and post-incubation with FCCP. Error bars = SEM, n ≥ 3, p values generated from two-tailed unpaired t-tests. Abbreviations: CI and V, complexes I and V; FCCP carbonyl cyanide 4-(trifluoromethoxy)phenylhydrazone; TMRM, tetramethylrhodamine methyl ester; $\Delta\Psi_m$, mitochondrial membrane potential.

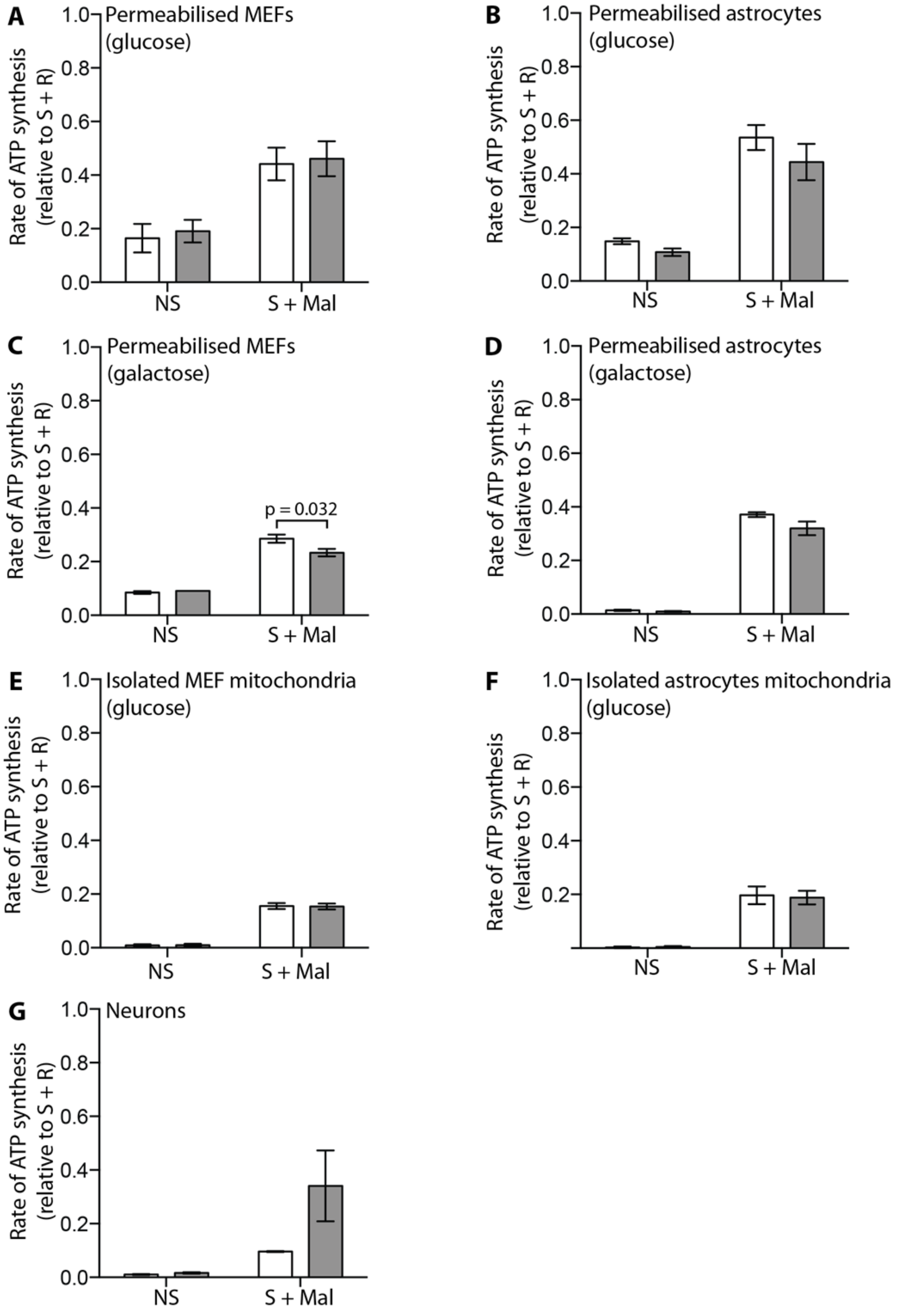
A
Permeabilised MEFs
(glucose)
B
Permeabilised astrocytes
(glucose)
C
Permeabilised MEFs
(galactose)
p = 0.032
D
Permeabilised astrocytes
(galactose)
E
Isolated MEF mitochondria
(glucose)
F
Isolated astrocytes mitochondria
(glucose)
G
Neurons
Rate of ATP synthesis
(relative to S + R)
1.0
0.8
0.6
0.4
0.2
0.0
NS
S + Mal

Figure S4. Control rates of ATP synthesis in *Ndufs4*$^{fky/fky}$ cell lines. The rates of ATP synthesis were measured in permeabilised cells or isolated mitochondria from MEFs and astrocytes maintained on glucose (A, B, E, F) or galactose (C, D), and neurons maintained on NBM medium (G) from +/+ (white bars) and *Ndufs4*$^{fky/fky}$ (grey bars) mice. The measurements were made using the substrate and inhibitor combinations indicated. Error bars = SEM. Replicates: NS ($n \geq 1$); and S + Mal ($n \geq 3$). p values generated from two-tailed unpaired t-tests. Abbreviations: Mal, malonate; NS, no substrate; S, succinate.
